# Electron paramagnetic resonance spectroscopy reveals alterations in the redox state of endogenous copper and iron complexes in photodynamic stress-induced ischemic mouse liver

**DOI:** 10.1016/j.redox.2020.101566

**Published:** 2020-05-12

**Authors:** Monika A. Jakubowska, Janusz Pyka, Dominika Michalczyk-Wetula, Krzysztof Baczyński, Maciej Cieśla, Anna Susz, Paweł E. Ferdek, Beata K. Płonka, Leszek Fiedor, Przemysław M. Płonka

**Affiliations:** a Małopolska Centre of Biotechnology, Jagiellonian University, Kraków, Poland; b Faculty of Biochemistry, Biophysics and Biotechnology, Jagiellonian University, Kraków, Poland; c Faculty of Chemistry, Jagiellonian University, Kraków, Poland

**Keywords:** Copper, EPR/ESR spectroscopy, Ischemia, Iron, Liver, Redox-active transition metals

## Abstract

Divalent copper and iron cations have been acknowledged for their catalytic roles in physiological processes critical for homeostasis maintenance. Being redox-active, these metals act as cofactors in the enzymatic reactions of electron transfer. However, under pathophysiological conditions, owing to their high redox potentials, they may exacerbate stress-induced injury. This could be particularly hazardous to the liver - the main body reservoir of these two metals. Surprisingly, the involvement of Cu and Fe in liver pathology still remains poorly understood. Hypoxic stress in the tissue may act as a stimulus that mobilizes these ions from their hepatic stores, aggravating the systemic injury. Since ischemia poses a serious complication in liver surgery (e.g. transplantation) we aimed to reveal the status of Cu and Fe via spectroscopic analysis of mouse ischemic liver tissue. Herein, we establish a novel non-surgical model of focal liver ischemia, achieved by applying light locally when a photosensitizer is administered systemically. Photodynamic treatment results in clear-cut areas of the ischemic hepatic tissue, as confirmed by ultrasound scans, mean velocity measurements, 3D modelling of vasculature and (immuno)histological analysis. For reference, we assessed the samples collected from the animals which developed transient systemic endotoxemic stress induced by a non-lethal dose of lipopolysaccharide. The electron paramagnetic resonance (EPR) spectra recorded *in situ* in the liver samples reveal a dramatic increase in the level of Cu adducts solely in the ischemic tissues. In contrast, other typical free radical components of the liver EPR spectra, such as reduced Riske clusters are not detected; these differences are not followed by changes in the blood EPR spectra. Taken together, our results suggest that local ischemic stress affects paramagnetic species containing redox-active metals. Moreover, because in our model hepatic vascular flow is impaired, these effects are only local (confined to the liver) and are not propagated systemically.

## Highlights

• Liver ischemia causes local dyshomeostasis in redox-active transition metal ions.
• Metal ion-reactive species interaction exacerbates injury of the hepatic tissue.
• Copper chelation could aid the removal of reactive species.

## Graphical abstract

**Figure:**
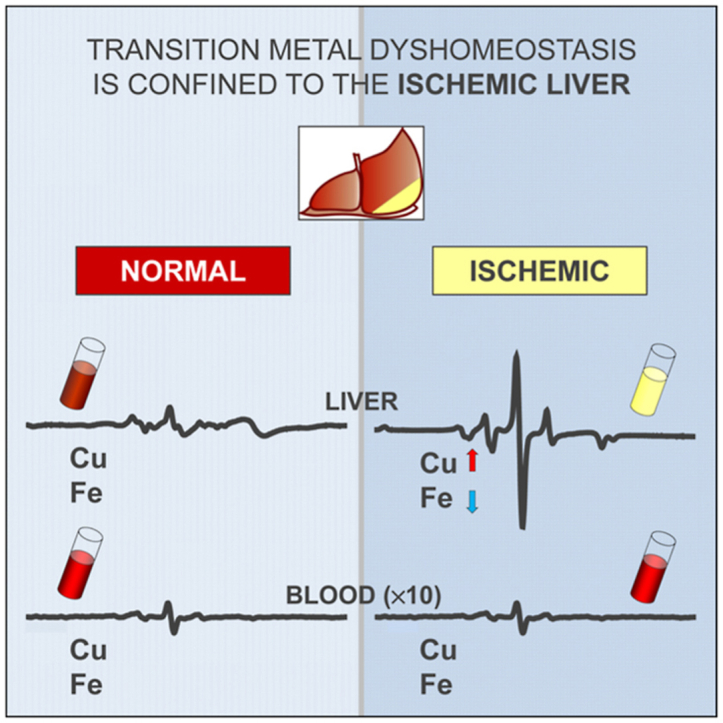

## Abbreviations

3D: three dimensional
α-SMA: alpha-smooth muscle actin
Ab: antibody
DAPI: 4′,6-diamidino-2-phenylindole
DETC: diethyldithiocarbamate
EPR/ESR: electron paramagnetic/spin resonance
Fe–S: the Riske cluster
HbNO: nitrosylhemoglobin
HE: hematoxylin and eosin
HFS: hyperfine structure
HRP: horseradish peroxidase
Ip: intraperitonealy
Iv: intravenously
LPS: lipopolysacharide
MetHb: methemoglobin
MGG: May-Grünwald-Giemsa
PBS(T): phosphate-buffered saline (Tween 20-supplemented)
PD: photodynamically
PDT: photodynamic therapy
RT: room temperature
Sc: subcutaneously
SEM: standard error of the mean
TAE: Tris-acetate-EDTA buffer
TBS: Tris-buffered saline
TF: transferrin
Zn-Pheide: zinc pheophorbide a

## 1 Introduction

Local circulation failure results in tissue restricted blood flow (hypoperfusion), followed by a shortage of nutrients and oxygen. In turn, a decreased level of oxygen (hypoxia) drives ischemic stress with further disruption in the tissue redox balance. This is particularly hazardous in organs with intensive oxygen metabolism such as the brain or the heart [1], where ischemic tissue injury may lead to necrotic cell death and development of infarction. Nevertheless, organs with somewhat lower oxygen requirements, such as the liver [1], may also suffer from hypoperfusion-related cellular damage, e.g. hypoxic hepatitis [2]. The pathogenesis of hepatic tissue injury could be even more complex since the liver serves as the main body reservoir of redox-active transition metal ions such as copper and iron [[3], [4], [5]]. Owing to their inherent flexibility in the oxidation state and the type and number of molecular bonds formed (ligancy), these metals act as static cofactors within protein active sites *in vivo* [6]. On the other hand, redox-active transition metal ions may catalyze the formation of harmful free radicals (e.g. lipid-based radicals) *in situ* in the cell. These reactive species are generated in the pathophysiological processes of oxidation/reduction fueled by the Fenton or Haber-Weiss reactions [[7], [8], [9]], predominantly in an environment of cellular membranes. In such a nonpolar environment the generation of lipid-based reactive species is dramatically accelerated by divalent metal ions, especially Fe(II) [10] and Cu(II) [11]. In order to prevent the potential metal toxicity from being exacerbated, the availability of redox-active transition metals is regulated in the cells. Significantly, recent studies on transition elements in biological systems have highlighted a regulatory role of iron in a type of cell death called ferroptosis, characterized by the accumulation of lipid peroxides [10,12]. However, the exact molecular mechanism that explains the role of Fe in this process has yet to be described [13].

In humans, both Cu and Fe are essential micronutrients, therefore a balanced supply of them in a diet is of critical importance for development and health. After intestinal absorption, these cations are transported to the liver, where they may be stored in hepatocytes as well as other cell types [14]. A normal human liver stores 15–55 μg Cu per 1 g of its dry weight [15], whereas its Fe content ranges from 0.2 to 2 mg per 1 g of hepatic dry weight [16]. These values vary between men and women and may depend on hormone levels (e.g. Fe content in the liver was found to be higher in post-menopausal women). Importantly, cases of Fe overdose in children have been reported after excessive intake of dietary supplements - brightly colored and sugar-coated, and thus often perceived as sweets by children [17]. What is more, the accumulation of redox-active transition metals can be elevated under certain genetic conditions that affect metal excretion by the liver cells. For instance, impaired release of Cu ions from the hepatocytes and thus its pathological accumulation in the liver results in severe organ toxicity implicated into the mechanism of Wilson's disease [4,5,18]. Another example of a metal-related pathology is hemochromatosis associated with excessive accumulation and toxicity of Fe, also in the liver [19]. However, the hepatotoxicity of copper/iron is not limited to sufferers of these relatively rare diseases. Redox-active transition metals have been reported to aggravate other liver pathologies such as ischemia/reperfusion injury [20], and others. Finally, as common environmental pollutants, Cu and/or Fe may affect a much larger number of people. Because of their accumulation in the mitochondrial membranes, they may induce peroxidation of membrane lipids, thus causing severe disturbances in respiratory chain and impairing normal mitochondrial functions [11,21]. This raises serious concerns over the adverse effects of both micronutrients we are exposed to in our daily diet, and the transition metal ions that have already accumulated in the liver. Normally being essential for good health, these metals, when in excess, may exacerbate certain pathophysiological conditions. In the case of ischemia/reperfusion injury, they could also affect the outcomes of life-saving therapies such as liver transplantation. Therefore, diagnosis based on a detailed description of the content of transition metal and their redox state *in situ* in the liver specimens would provide data of predictive and/or prognostic value. This could be achieved by the use of electron paramagnetic/spin resonance (EPR/ESR) spectroscopy, which detects the paramagnetic species characterized by the presence of unpaired electrons. EPR spectroscopy may also be used to draw conclusions regarding non-metal paramagnetic molecules such as NO, an inflammatory mediator and blood flow regulator produced by nitric oxide synthases (NOS).

Importantly, transition metal ions may bind to specific sites on NOS, and thus affect its enzymatic activity [22]; however, among the metals that have been studied only non-heme iron increases NOS activity [22]. Other divalent transition metals are unable to activate NOS; in fact, for Cu(II), an inhibitory effect has been demonstrated [22]. These interactions with NOS, however, might be perceived as a double-edge sword. NO is a very potent vasodilator, which, when generated locally, facilitates vascular drainage of toxic species produced when the tissue is damaged [23]. Since ischemic liver injury affects hepatic microvasculature and impairs the function of endothelial NOS (eNOS), it causes a decline in the local production of NO [23]. In such a case, the inhibitory effect of transition metals on eNOS becomes even more pronounced, further exacerbating tissue damage. On the other hand, upon restoration of blood flow, NO reacts with oxygen, which yields toxic reactive nitrogen species, resulting in the reperfusion injury. However, the detailed mechanisms of *in vivo* NO-metal ion interactions, and their impact on human health, are yet to be described. This has prompted us to investigate NO-redox-active transition metal tissue homeostasis in the normal versus pathological liver and in the blood, in animal model. Two mouse models of liver stress were used: (i) the photodynamic stress model developed by us [24], and (ii) the endotoxemic stress model. In the former model, a light-activated pro-drug is administered systematically, after which red light is applied locally (for localized excitation of the pro-drug) in order to initiate the production of reactive species *in situ.* This yields a clearly defined focal area of pale ischemic hepatic tissue, in which the organ parenchyma and vasculature are affected by photodynamically-generated toxic species. The same principle underlies photodynamic therapy (PDT), which can be employed to target solid tumors [25] or microbial skin lesions [26]. In the latter approach, endotoxemic stress is induced by systemically administering a non-lethal dose of lipopolysaccharide (LPS), which elicits a temporary (up to 24 h) systemic endotoxemic shock in the animals undergoing this procedure [27]. We used noninvasive organ imaging of the live animals along with a detailed microscopic and spectroscopic analysis of tissue specimens to assess the involvement of redox-active transition metals and other paramagnetic species in liver stress. First, abdominal ultrasound imaging (USG) was employed to estimate the extent of ischemic damage in the hepatic parenchyma/vasculature, further supported by measurements of the mean velocity and computational 3D modelling of the liver vasculature. Then, (immuno)histological analysis of the tissue sections was used to characterize those cell populations infiltrating the ischemic areas. Finally, EPR spectroscopy and spin trapping were used to reveal the characteristic “fingerprints” of the paramagnetic centers recorded in the injured and control livers.

## 2 Materials and methods

### 2.1 Materials

#### 2.1.1 Animals

C57BL/6J mice (male, 7 weeks old) were purchased from the Mossakowski Medical Research Center of the Polish Academy of Sciences (Warsaw, Poland), or were obtained from the local animal facility of the Faculty of Biochemistry, Biophysics and Biotechnology, the Jagiellonian University (Kraków, Poland). The mice were kept in a 12/12 h light/dark cycle, with free access to food (standard laboratory rodent chow) and water.

#### 2.1.2 Photosensitizer

Zinc pheophorbide *a* (Zn-Pheide) was obtained as described previously [24,25,28,29]. Briefly, chlorophyll *a* was extracted from *Spirullina laporte* and treated with chlorophyllase (EC No. 3.1.1.14) to remove the hydrophobic phytyl moiety [28]. The product was first demetalated using glacial acetic acid and then metalated directly with zinc acetate. After purification, Zn-Pheide was stored at −30°C under Ar atmosphere. Zn-Pheide is a very potent singlet oxygen photosensitizer, as indicated by our earlier *in vitro* and *in vivo* studies [24,25]. Owing to the presence of a central Zn ion, this photosensitizer produces ^1^O_2_ with a very high quantum yield of ~0.75. This is much higher ΦΔ than those of the unmodified chlorophylls [30]. Our recent study [31] has shed more light on the photochemical properties of Zn-Pheide: we have shown that more than 90% of the first excited triplet state of this photosensitizer yields ^1^O_2_. This translates into practically entire photosensitization via Type II reaction that is mediated by ^1^O_2_ [31]; therefore, in our system, free radicals (Type I reaction) are not expected to be generated in any significant amounts.

#### 2.1.3 Selected chemicals, drugs and antibodies

Accustain May-Grünwald stain, ammonium chloride, bovine serum albumin (BSA), eosin Y, ferrous sulfate (FeSO_4_ × 7H_2_O), Giemsa stain, lipopolysaccharide (LPS) from *E. coli* serotype O26:B6, sodium citrate (C_6_H_5_Na_3_O_7_ × 2H_2_O), sodium diethyldithiocarbamate (C_5_H_10_NS_2_Na), Sudan Black B, TAE buffer, TBS buffer, and Triton X-100 were purchased from Sigma-Aldrich (St. Louis, US); ammonia water from POCh (Gliwice, Poland); hematoxylin from Aqua-Med (Łódź, Poland); Aqua Mount, Consul Mount, paraffin from Thermo (Waltham, US); Polysine and Superfrost Slides from Gerhard Menzel GmbH (Brunswick, Germany); Tween 20 from ICN Biomedicals (Irvine, US); ProLong Diamond Antifade Mountant with DAPI from Fisher Scientific (Loughborough, UK); streptavidin-HRP from Dako (Carpinteria, US); DAB substrate peroxidase kit from Vector Laboratories (Burlingame, US); isoflurane (*AErrane*) from Baxter Pharmaceutical Products (Deerfield, US); ketamine (*Bioketan*) and xylazine (*Sedazin*) from Vetoquinol Biowet (Puławy, Poland); medetomide hydrochloride (*Cepetor*) from ScanVet (Gniezno, Poland); Aquasonic CLEAR ultrasound gel and SIGNAGEL electrode gel from Parker Laboratories (Fairfield, US); and Veet depilatory cream from Reckitt Benckiser (Nowy Dwór Mazowiecki, Poland). Primary and secondary antibodies (Abs) used in this study are listed in Table 1.

**Table 1:** Antibodies used for immunohistochemical studies.

Primary antibody	Species	Manufacturer (Catalog No.)
Anti-α-SMA	Mouse monoclonal	Abcam (7817)
Anti-CD45	Rat monoclonal	Novus Biological (100-77417SS)
Anti-CD31	Rat	BD Pharmingen (550274)
Anti-CD107b	Rat	BD Pharmingen (550292)

Secondary antibody	Species	Manufacturer (Catalog No.)

AlexaFluor 488 anti-rat	Goat	Fisher Scientific (10694383)
AlexaFluor 635 anti-mouse	Goat	Fisher Scientific (10413412)
Biotin anti-rat Ig	Goat	BD Pharmingen (559286)
Biotin anti-rat IgG1/2a	Mouse	BD Pharmingen (550325)

Abcam, Cambridge, UK; BD Pharmingen, San Jose, US; Novus Biologicals, Cambridge, UK; Fisher Scientific (Loughborough, UK).

## 3 Methods

### 3.1 Ethical aspects of the studies

The *in vivo* studies were carried out at the Jagiellonian University (Kraków, Poland), and all the experimental procedures and approaches were approved by the Local Ethical Committee for Animal Experimentation (Kraków, Poland; decisions No. 46/2012 and 60–62/2013), in accordance with EU Directive 2010/63/EU for animal experiments. The animals received humane care and were allowed to settle for two weeks before the experiments; the volumes of liquid administered intraperitoneally (*ip*) did not exceed 0.5 ml, intravenously (*iv*) 0.2 ml, and subcutaneously (*sc*) 0.05 ml.

### 3.2 Study design: photodynamic and endotoxemic models of liver stress

**Fig. 1:**
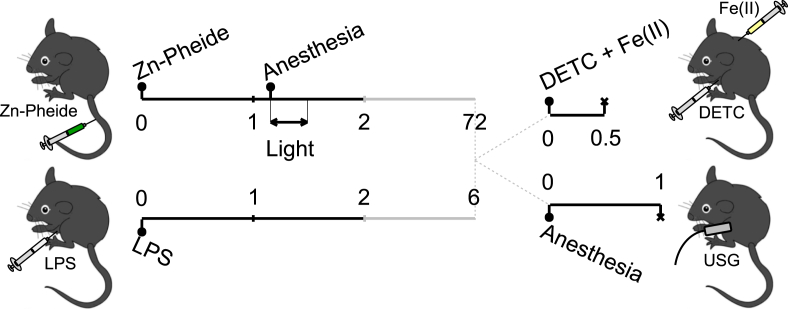
Two models of liver stress - summary of the experiments. C57BL/6J mice were challenged either phodynamically or endotoxemically (PD- or LPS-challenge, respectively), or remained unchallenged (controls). Left schemes show the treatment. *PD-challenge:* Mice received *iv* injections of a light-activated pro-drug Zn-Pheide and were then illuminated with red light; and *LPS-challenge:* Mice received *ip* injections of LPS. Right schemes show data acquisition. *EPR spin trapping:* Mice received *ip* injections of DETC and subsequent *sc* injections of Fe(II) complexes (forming together the EPR spin traps). Then the mice were humanely killed, and the blood and the liver samples were collected; the livers were either fixed for (immuno)histological analysis, or were snap frozen in liquid nitrogen along with the blood samples, and EPR spectra of the samples were recorded; *USG-Doppler analysis:* Mice received isoflurane inhalation (anesthetic) and anatomical images of the liver tissue along with the mean velocity maps were recorded. Black solid lines: continuous time scale [h]; grey solid lines: time scales [h] 2–72 for PD- and 2–6 for LPS-challenge groups, respectively; grey dashed lines: transitions from treatment to data acquisition; filled circles (•): injection/inhalation points; and multiplication signs (×): experimental end-points.

The experimental design and time scheme are summarized in Fig. 1. The model of local photosensitizer-and-light-induced stress (PD-challenge) in a mouse liver was introduced in our previous work [24]. Briefly, the animals were treated with Zn-Pheide (2 mg/kg, *iv*) 70 min before illumination with monochromatic 655 nm red light emitted by a diode laser (Eurotek, Warsaw, Poland). Light was applied for 20 min at a fluency of 100 mW/cm^2^, reaching a total light dose of 120 J/cm^2^. Before illumination, the animals were anesthetized by *ip* application of ketamine (8 mg/kg) and medetomide hydrochloride (0.1 mg/kg), then the hair was removed from approximately 1 cm^2^ of their left thoracic areas and the animals were placed on a 38°C-heating platform and illuminated. Untreated animals as well as the animals that received light only- or Zn-Pheide only-treatment were used as the control groups. Endotoxemic stress was induced by a single injection of lipopolysaccharide at a non-lethal dose (10 mg/kg, *ip*) which resulted in systemic shock (LPS-challenge) [27].

### 3.3 Ultrasound imaging and mean velocity measurements

Noninvasive medical ultrasound imaging of the liver tissue was carried out and the mean velocity in the hepatic vasculature was assessed using a Vevo 2100 high-frequency, high-resolution digital imaging platform (VisualSonics, Toronto, Canada). The data were collected from the untreated control, and from the LPS- or PD-challenged animals, with n=3 mice per group. The animals were anesthetized with isoflurane (2%, 0.5 L/min) and restrained on a 38°C-heating platform. Their paws were covered with SIGNAGEL electrode gel and approximately 1 cm^2^ of their left thoracic areas were depilated using a cream. The hair-free areas were then covered with Aquasonic CLEAR ultrasound gel, the ultrasound transducer was transferred to the 3D motor stage and the 30 MHz respiratory-gated signals were collected from the livers using a 3D-Mode imaging option. At the same time, a Power Doppler Mode imaging option was used to register the mean velocity of the blood; in parallel, the animal heartbeats were monitored using electrocardiography. After imaging, the animals were humanely killed by isoflurane overdose. All images were recorded digitally, and they were analyzed using Vevo 2100 1.2.0 software and the methods described below.

### 3.4 Computational models of the liver vasculature

Original Vevo 2100 *dmc* files were converted to *png* files using dcm2pnm 3.5.4 (Kuratorium OFFIS, Oldenburg, Germany), and then the Python Image Library 1.1.7 (Secret Labs, Linköping, Sweden) was used to process the images further, such as to crop them (remove the outer parts of an image in order to improve framing), posterize (convert a continuous gradation of tone to several regions of fewer tones) and reduce the image color palette. The volume was visualized by processing the data with VolView 3.4 (Kitware, New York, US), and then the 3D models obtained were converted into a mesh format by Paraview 4.0.1 (Kitware, New York, US). The mesh-transformed models were processed using MeshLab 1.2.2 (ISTI-CNR, Pisa, Italy): the noise, originating from either the skin or thoracic diaphragm vasculature was removed, the number of vertices (the points where two lines meet to form an angle) was reduced and the unwanted boundary image fronts deleted. Finally, Blender 2.67b (Stichting Blender Foundation, Amsterdam, The Netherlands) was used to visualize the models.

### 3.5 Tissue analysis

*Histology:* The liver sections were stained using hematoxylin and eosin (HE) and May-Grünwald-Giemsa (MGG) as described previously [32].

*Immunohistochemistry - DAB-HRP detection:* The 6 μm frozen liver sections were mounted on Superfrost glass slides, allowed to dry at room temperature (RT; 1 h); then they were dehydrated in cold acetone (10 min), dried again (RT; 30 min), and stored in phosphate-buffered saline (PBS) at 4°C until further use for staining. The sections were washed (5 min, ×2) in 0.04% Tween 20 in PBS (PBST) containing 0.025% Triton X-100, followed by blocking in 10% fat-free milk supplemented with 1% bovine serum albumin (10 min), and then in egg white (10 min). These and the following steps were carried out in a humid chamber. Then the sections were incubated with primary rat anti-mouse CD107b (0.6 μg/ml; 1 h) or rat anti-mouse CD31 (1.6 μg/ml; 1 h) antibodies (Abs) prepared in PBST containing 0.025% Triton X-100, and washed (5 min, ×2). The endogenous peroxidases were inhibited by 3% hydrogen peroxide (15 min); the sections were then washed (3 min), incubated with secondary biotin mouse anti-rat (2.5 μg/ml; 1 h) or biotin goat anti-rat (2.5 μg/ml; 1 h) Abs, and washed (5 min, ×3). They were then incubated with streptavidin-conjugated horseradish peroxidase (HRP, 30 min), washed (3 min), and incubated with 3,3′-diaminobenzidine (2 min); Ni-enhancement was additionally used for the CD107b staining. Chromogen development was stopped by washing with tap water (5 min). The sections were counterstained in Mayer's hematoxylin (3 min), washed with tap water (3 min) and in 0.2% ammonia water, and were sealed in Aqua-Mount mounting medium. The tissue slides were inspected under a light microscope Eclipse Ti (Nikon, Tokyo, Japan), photographed and analyzed. The list of Abs used is given in Table 1.

*Immunohistochemistry - fluorescence detection:* The procedure was performed as described previously [33]; the primary Abs were mouse monoclonal anti-alpha-smooth muscle actin (α-SMA; 3.3 μg/ml; 1 h) and rat monoclonal anti-CD45 (1 μg/ml; 1 h). The negative controls were incubated in blocking buffer with no primary Abs. The secondary Abs were AlexaFluor 635 goat anti-mouse (4 μg/ml; 1 h) and AlexaFluor 488 goat anti-rat (4 μg/ml; 1 h), applied consecutively and separated by washing. The tissue was preserved in antifade mounting medium with DAPI. The slides were imaged using a two-photon TCS SP5 II confocal microscope (Leica, Wetzlar, Germany), excitation wavelengths: 355, 488, 633 nm; laser power: 60, 15 and 4%, respectively; resolution 512 × 512) and dry 20× HC PL FLUOTAR objective, and were then stored at 4°C. The brightness and contrast in the confocal images were adjusted using software supplied by the manufacturer. The list of Abs used is given in Table 1.

### 3.6 Electron paramagnetic/spin resonance (EPR/ESR) measurements

*Spin trap administration and sample preparation:* Summary of the experiments is presented in Fig. 1; the reagents were prepared according to the protocols described previously [27]. The spin trap was administered to all experimental animals (with exception of the “No spin trap” group) in a classical two-step fashion [[34], [35], [36], [37]]. First, mice received (i) a single *ip* injection of DETC (500 mg/kg), and then (ii) a subsequent *sc* injection of chelated iron(II) complexes of ferrous sulfate together with sodium citrate (50 mg/kg and 250 mg/kg, respectively) into the scruff of the neck [36]. This intentional spatial separation of spin trap components - at the moment of injection - prevented an insoluble precipitate from being formed in the animal cavity fluids [[34], [35], [36],^38^]. By adopting this strategy in our experiments, we provided means of the efficient and safe spin trap delivery. 30 min after the administration of both spin trap components, they had fully combined, yielding an active spin trap in the animal organs/blood [27]. The mice were then deeply anesthetized (ketamine and xylazine, 150 mg/kg and 10 mg/kg, *ip*) and the blood was collected from the beating hearts. The livers were then dissected, photographed and the edge parts of *lobus* *hepatis* *sinister lateralis*, along with blood aliquots, were snap frozen in liquid nitrogen in 2 cm long glass tubes (inner diameter 0.4 cm), and then were stored at −80°C until spectroscopic analysis [24].

*EPR spectroscopy:* The samples were measured at 77 K in a cold finger quartz Dewar using an EPR X-band (ca. 9.4 GHz) spectrometer EMX (Bruker BioSpin, Rheinstetten, Germany). The following parameters were applied: field 333.5 ± 25 mT (signals from the g = 2.0 region) or 133.5 ± 60 mT (signals from the g = 4.3 or g = 6.0 regions), modulation amplitude 0.5 mT, conversion time 40.96 ms, time constant 20.48 ms, microwave power 4.2 mW, receiver gain 1 × 10^4^ (liver) or 1 × 10^3^ (blood), and resolution 1024 points. A total number of 16 scans were accumulated in each spectrum. The spectra were analyzed using WinEPR software (Bruker BioSpin, Rheinstetten, Germany) and Eleana 0.9.8, free software developed by Dr. Marcin Sarewicz and other contributors (Department of Molecular Biophysics, the Jagiellonian University, Kraków, Poland; see https://eleana.larida.pl/). Eleana software offers a “Read position” mode that returns an exact g value for any magnetic induction of interest within the measured range. We used this software function to read the analytical g values, and then, on the basis of the literature data, to identify the paramagnetic components in the liver and blood spectra. The characteristics of the measured EPR signals along with the references are summarized in Table 2.

**Table 2:** Characteristics of the EPR (ESR) signals measured - a summary of the literature data.

Paramagnetic signal		*g*-factor literature value	HFS (analytical lines)	References
LIVER				
NO–Fe^II^-(DETC)_2_	Iron(II) diethyldithiocarbamate complexes	2.035	Triplet (III)	[36,59,65]
Cu^II^(DETC)_2_	Copper(II) diethydithiocarbamate complexes	2.025	Quarter (III)	[36,61]
Semiquinone	Semiquinone free radical*	2.004	–	[65]
Mo(V)	Molybdenum(V)*	1.97	–	[66]
Fe–S	The reduced Riske cluster*	1.94	–	[59,[66], [67], [68]]
BLOOD				
MetHb	Methemoglobin	6.0	–	[63,67]
Fe^III^-TF	Transferrin-bound iron(III)	4.3	–	[59,63]
Cu^II^(DETC)_2_	Copper(II) diethyldithiocarbamate complexes	2.025	Quarter (III, IV)	[36,61]
HbNO	Nitrosylhemoglobin	2.012	Triplet (I, III)	[[69], [70], [71]]

EPR(ESR), electron paramagnetic (spin) resonance; HFS, hyperfine structure; I, II, etc., lines I, II, etc. (low to high-field) of the hyperfine structure; *these are the co-factors of xanthine oxidase [72].

### 3.7 Statistical analysis

The quantitative results were presented as the mean ± standard error of the mean (SEM). For the statistical analysis, the Mann-Whitney *U* test was used, and the *p* values < 0.05 were considered significant.

## 4 Results

**Fig. 2:**
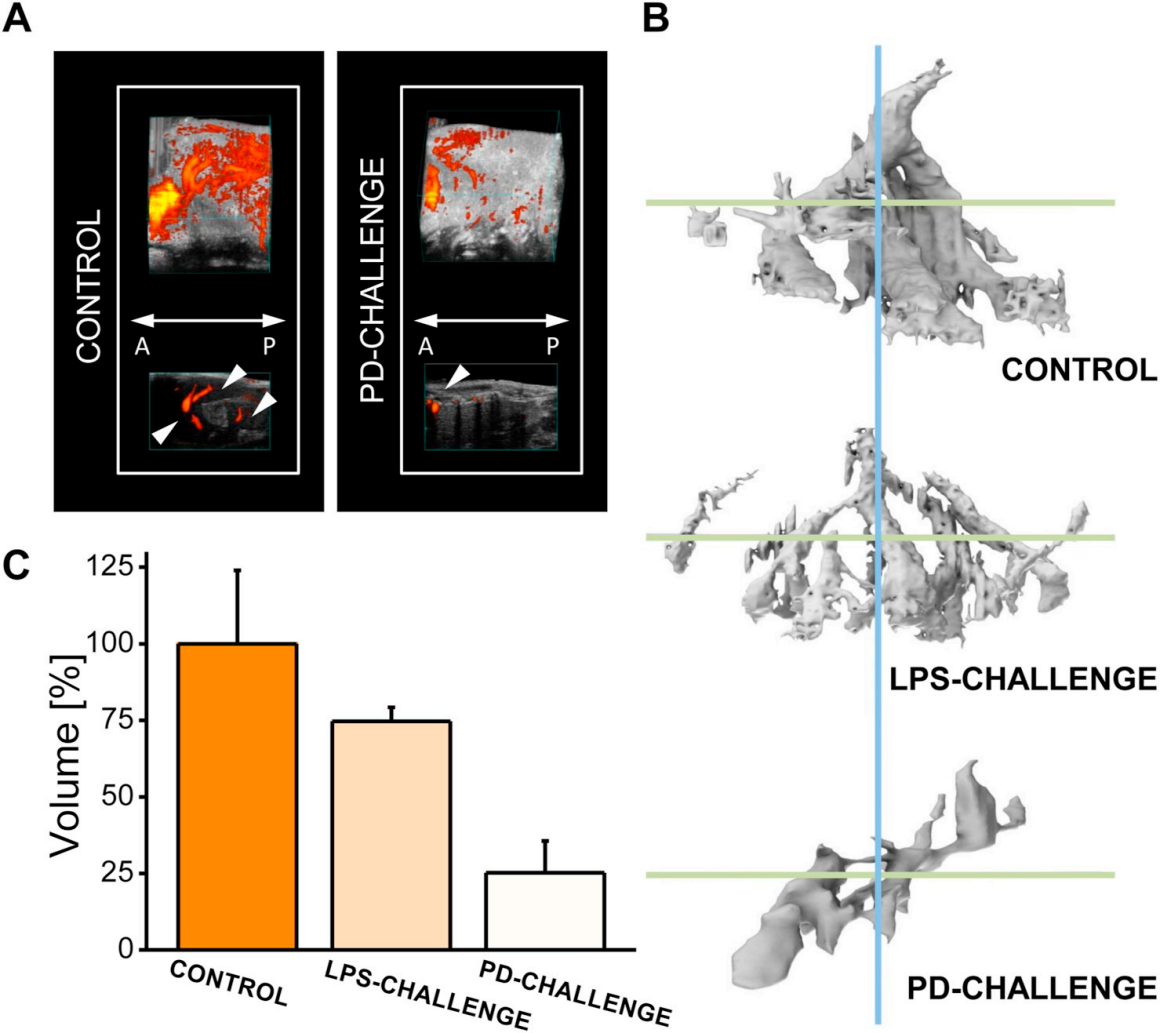
Photodynamically-induced stress affects the liver vasculature and blood flow. **A** Representative sagittal plane images of the control (left) and PD-challenged (right) livers. The series of liver images were collected in a 3D-imaging mode (black and white anatomical images) combined with a Power Doppler mode (color-coded mean velocity maps, in which yellow represents the highest and red the lowest speed of blood flow). A: anterior (the front); and P: posterior (the back) of an animal; white triangles: blood vessels, n=3 mice per group. **B** Representative 3D-reconstruction images of the liver vasculature - computational models of the control (upper), LPS-challenged (middle) and PD-challenged (bottom) livers. Blue line: sagittal axis; green lines: frontal axes. **C** Functional vasculature volume of the control (orange), LPS-challenged (yellow) and PD-challenged (cream) livers. n=3 mice per group, results presented as the mean % ± SEM.

**Fig. 3:**
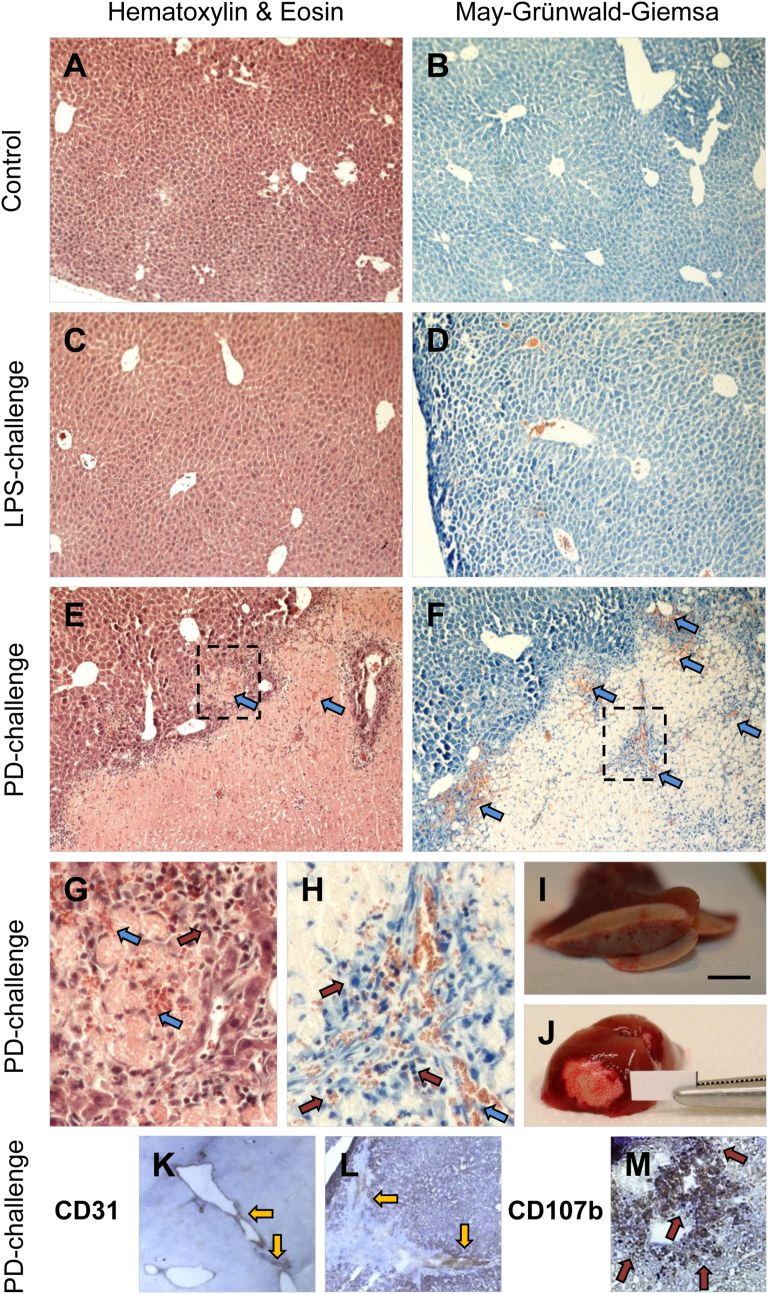
Photodynamically-induced stress causes ischemic necrosis in the liver. Control (**A-B**), LPS-challenged (**C-D**) and PD-challenged (**E-H**) livers were stained using hematoxylin and eosin (**A, C, E, G**) or May-Grünwald-Giemsa (**B, D, F, H**) staining methods; paraffin-embedded tissue slices, original magnification: 100×, brightness and contrast improved. **A-B** Control tissues show normal hepatic morphology. **C-D** LPS-challenged livers show similar morphology to the control. **E-F** PD-challenged livers contain both the areas of normal tissue (left corners purple/dark blue) and the areas of ischemic necrosis (right corners pale pink/light blue); leukocyte infiltration can be seen at the border between these areas. Blue arrows show the presence of extravasated blood. **G-H** Expanded areas shown in **E-F** with dashed-line rectangles. An islet of ischemic tissue surrounded by normal tissue (**G**) and the area around a collapsed blood vessel (**H;** see also Fig. 3L); original magnification: 120×. Blue arrows: extravasated blood; red arrows: leukocyte infiltration. **I-J** Macro photographs of PD-challenged livers. Yellow color indicates ischemic necrosis. Scale: 1 cm. **K-M** PD-challenged livers were stained immunohistochemically using anti-CD31 (**K-L**) or anti-CD107b (**M**) antibodies; frozen tissue slices, DAB-HRP staining (with Ni signal-enhancement in **M**), nuclei counterstained with hematoxylin, original magnification: 120×, brightness and contrast improved. **K-L** Vascular protein CD31 visualized in the normal (**K**) and the collapsed blood vessels (**L;** see also Fig. 3H) of a PD-challenged liver. Yellow arrows: staining (brown) of CD31. **M** CD107b protein, expressed by mononuclear phagocytes, is visualized in the tissue surrounding two non-collapsed blood vessels in the ischemic part of a PD-challenged liver. Red arrows show the staining (dark brown to black) of the CD107b-positive immune cells infiltrating the tissue.

**Fig. 4:**
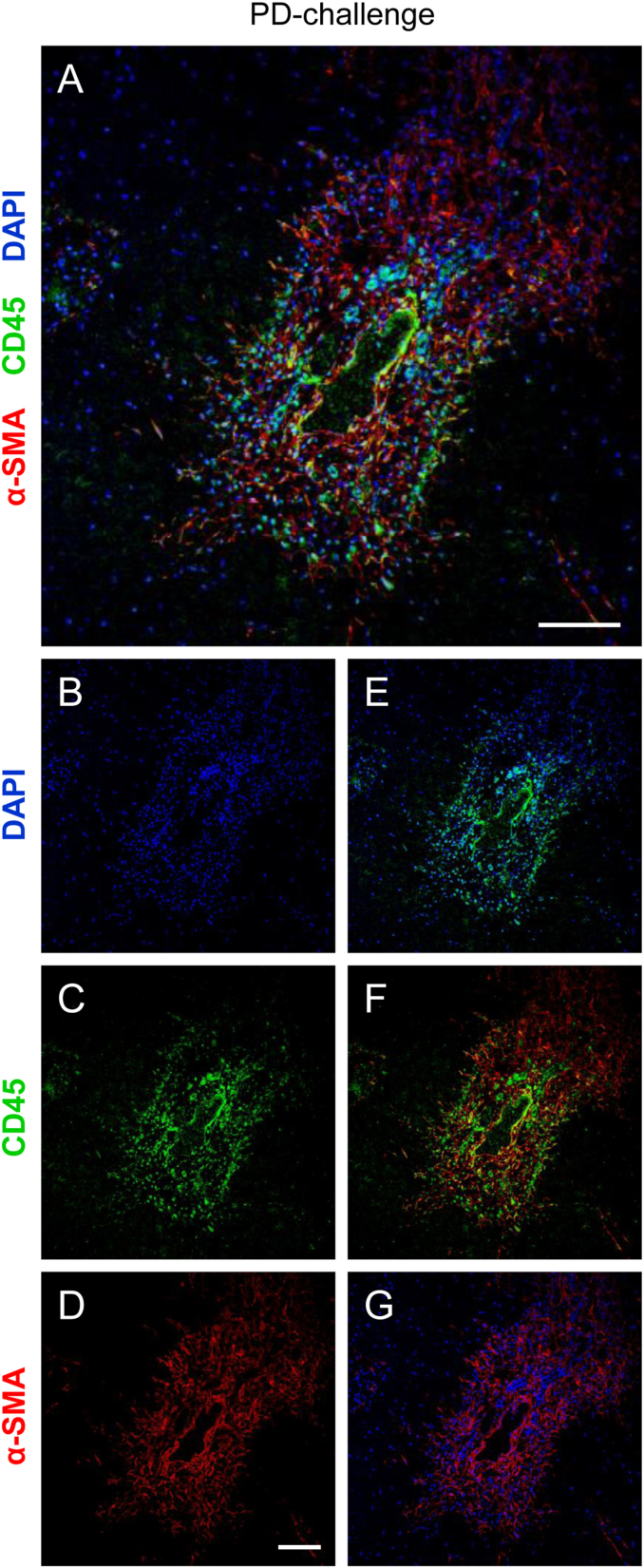
The boundaries between the scar and normal tissue are the areas of intensive reactive and regenerative processes. **A** PD-challenged livers were stained immunohistochemically using anti-CD45 (green) together with anti-α-SMA (red) antibodies; paraffin-embedded tissue sections, nuclei counterstained with DAPI (blue), original magnification: 20× , scale bar: 100 μm, brightness and contrast improved. **B-D** Single channel-components of an image **A;** nuclei stained with DAPI; blue (**B**), the pan-leukocyte protein CD45-expressing cells infiltrate the scar; green (**C**), and the α-SMA-expressing mesenchymal cells form the network that surrounds a non-collapsed blood vessel, spreading toward the necrotic tissue; red (**D**). **E-G** each overlays two channels shown in **B-D;** blue and green (**E**), green and red (**F**), and blue and red (**G**). **B-G** scale bar: 100 μm. See also Fig. 3H, L-M.

**Fig. 5:**
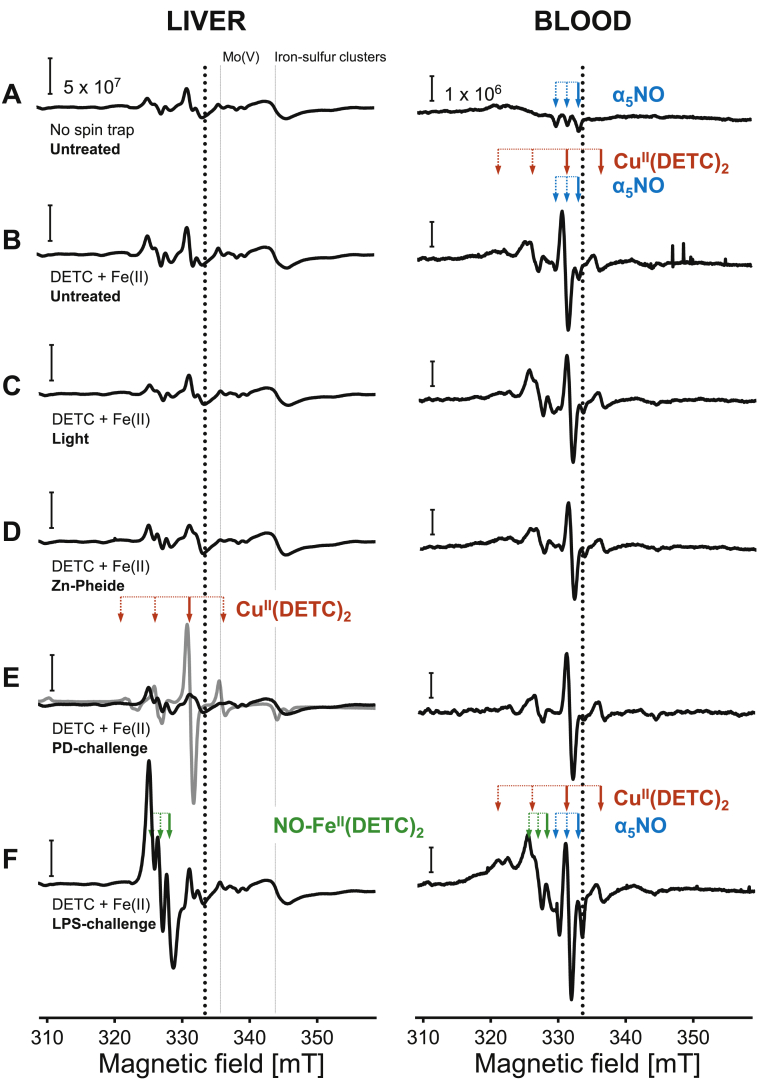
Both photodynamic and endotoxemic stress impact the liver free radical spectral profiles. Representative EPR spectra recorded *in situ* in the liver (left) and whole blood (right) samples collected from the control (**A-D**) or the challenged (**E-F**) animals. **A** Spectra of spin trap-free samples. **B–F** Fe^II^-DETC complexes served as the exogenous spin traps in the vehicle (**B**), vehicle and light (**C**) and Zn-Pheide and no light (**D**) control groups; and in the photodynamically (**E**) or endotoxemically-challenged (**F**) groups; scale: 5 × 10^7^ (left) and 1 × 10^6^ (right), normalized amplitude [a.u./g]. Thick black dotted lines: center of the magnetic field, at g = 2.0; thin black dotted lines (left to right): molybdenum(V) and reduced iron-sulfur cluster signals in the liver; black solid lines: normal liver/blood spectra; grey solid line: ischemic liver spectrum; dotted/solid horizontal lines with the arrows - the hyperfine structure (HFS) markers: α_5_NO, blue, NO–Fe^II^(DETC)_2_, red, and Cu^II^(DETC)_2_, green; note that the thick solid arrows indicate the analytical amplitudes. See also Table 2.

**Fig. 6:**
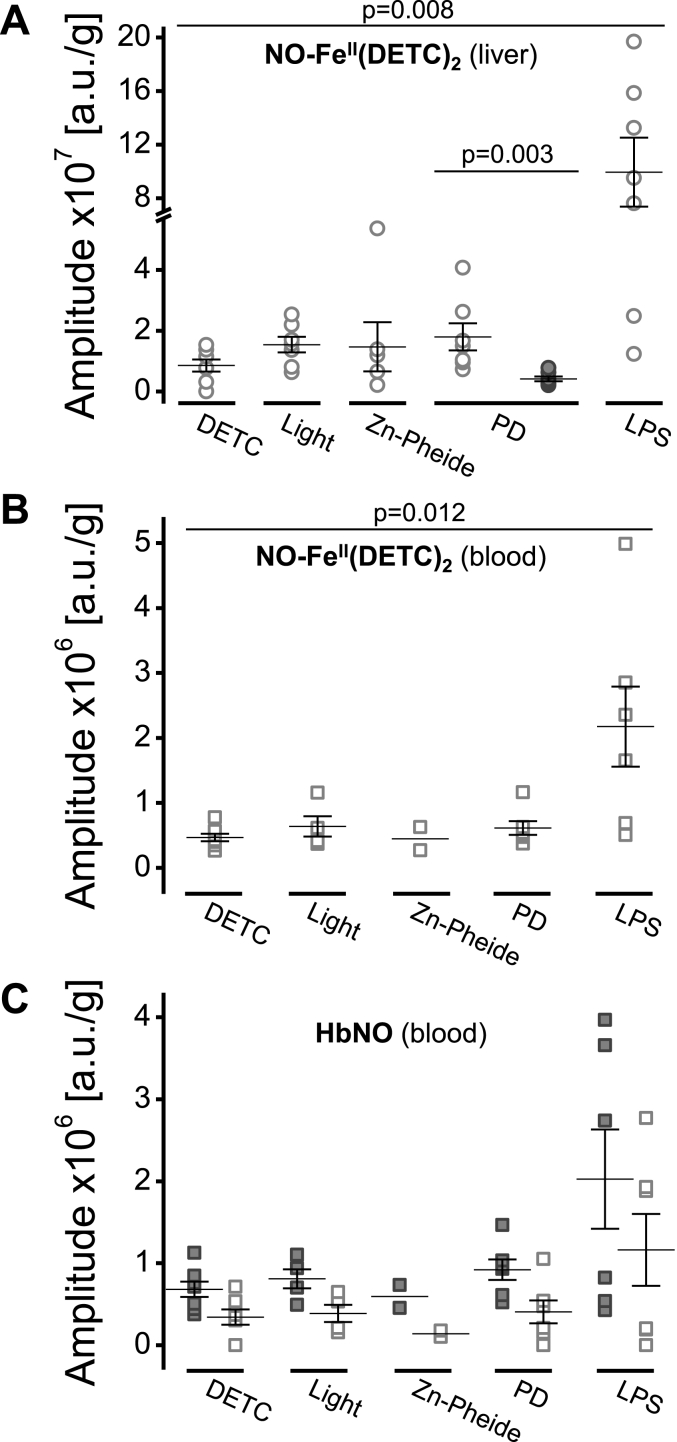
Elevated liver and blood nitric oxide levels are both associated with stress. **A-C** Normalized amplitudes [a.u./g] of the EPR signals recorded in the liver (**A**) and whole blood (**B–C**) samples collected from the control (DETC, Light and Zn-Pheide) or the challenged (PD and LPS) animals. Results are presented as individual data points and the mean ± SEM; *p* values (the Mann-Whitney *U* test) indicate significant differences between the groups. **A** Amplitudes of NO–Fe^II^(DETC)_2_ signals, line III, in the normal (empty circles) or ischemic (filled circles) liver tissue. Break: 5.7 ÷ 7.0. **B** Amplitudes of NO–Fe^II^(DETC)_2_ signals, line III, in the blood. **C** Amplitudes of HbNO signals, line I (empty squares) and line III (filled squares), in the blood.

**Fig. 7:**
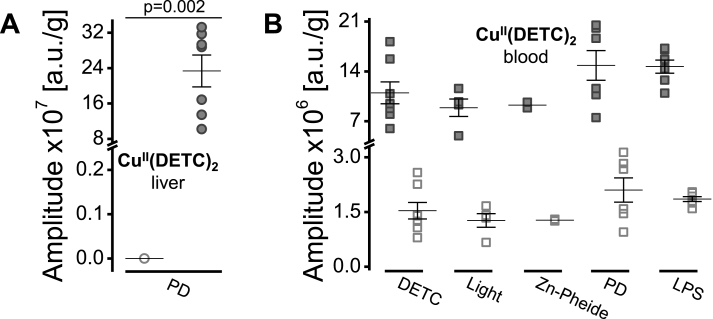
The copper(II) content is substantially higher in the ischemic tissue compared to the normal liver. Normalized amplitudes [a.u./g] of the EPR signals recorded in the liver (**A**) and whole blood (**B**) samples collected from the control (DETC, Light and Zn-Pheide) or the challenged (PD and LPS) animals. Results presented as individual data points and/or the mean ± SEM; *p* values (the Mann-Whitney *U* test) indicate significant differences between the groups. **A** Amplitudes of Cu^II^(DETC)_2_ signals in the normal (empty circles) or ischemic (filled circles) liver tissue. Break: 0.25 ÷ 7.0. **B** Amplitudes of Cu^II^(DETC)_2_ signals in the blood; line III (filled squares) and line IV (empty squares). Break: 3.3 ÷ 4.1.

**Fig. 8:**
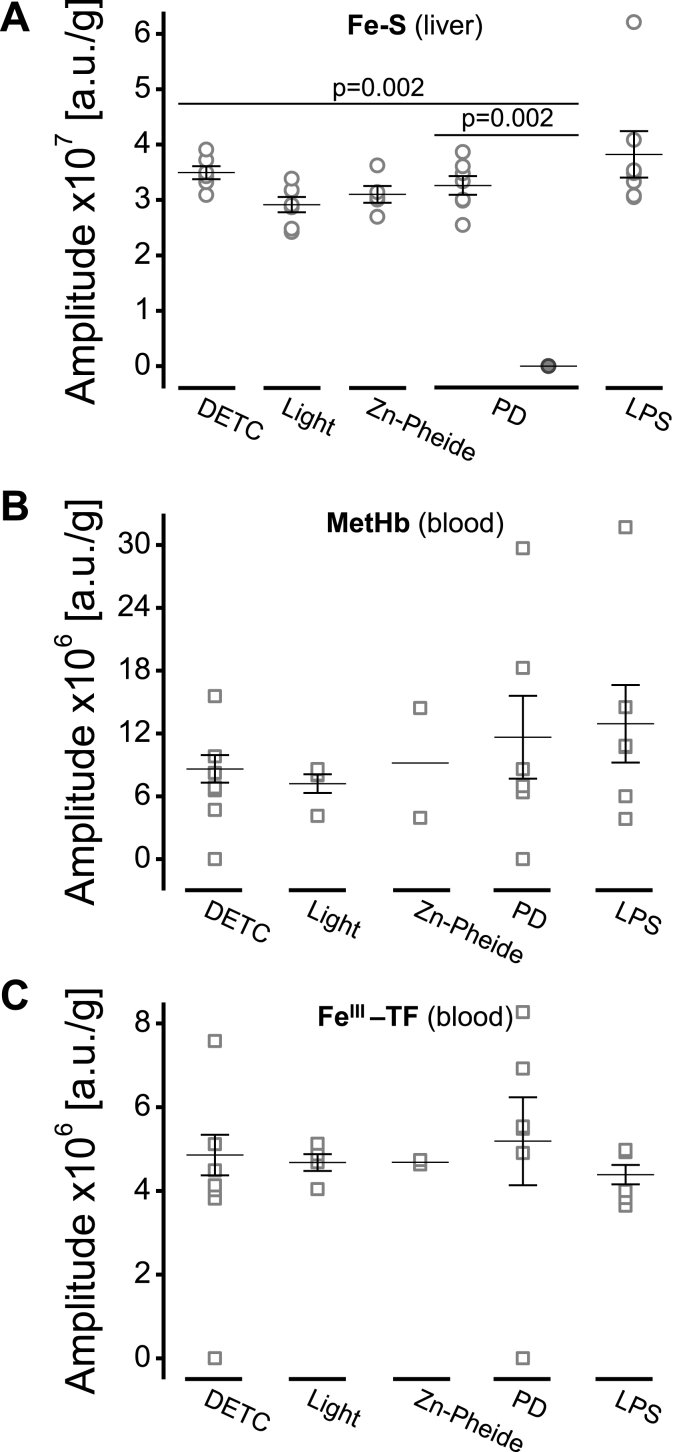
Photodynamically-induced stress affects the iron content in the ischemic liver and causes Fe elevation in blood. Normalized amplitudes [a.u./g] of the EPR signals recorded in the liver (**A**) and whole blood (**B–C**) samples collected from the control (DETC, Light and Zn-Pheide) or the challenged (PD and LPS) animals. Results presented as individual data points and the mean ± SEM; *p* values (the Mann-Whitney *U* test) indicate significant differences between the groups. **A** Amplitudes of the reduced Riske cluster signals in the normal (empty circles) or ischemic (filled circles) liver tissue. **B** Amplitudes of methemoglobin signals in the blood. **C** Amplitudes of Fe^III^-transferrin signals in the blood.

Two experimental models of photodynamically- or endotoxin-induced stress in the mouse liver (PD- or LPS-challenge, respectively; Fig. 1) were studied, in which blood flow (Fig. 2), tissue morphology (Fig. 2, Fig. 3, Fig. 4), protein expression (Fig. 3, Fig. 4), and EPR signals (Fig. 5, Fig. 6, Fig. 7, Fig. 8) were analyzed. Importantly, no symptoms of challenge-induced jaundice (such as a yellow-coloration of the skin, mouth or mucous membranes) were found in either the control or the experimental animals (not shown); no color changes in the urine/feces were present. This confirms that in these livers at least some parts maintained their physiological functions, including the production and secretion of bile.

### 4.1 Photodynamic but not endotoxin challenge affects the liver tissue and reduces blood flow in hepatic vasculature

We first analyzed the liver tissue and hepatic vasculature using the non-invasive methods of small-animal imaging. The ultrasound examination (USG) along with an assessment of the mean velocity (speed of blood flow) in this organ revealed the differences between the PD- or LPS-challenged versus control mice (Fig. 2). The black and white anatomical images demonstrated the presence of areas of high echogenicity (depicted in light greys in the image) in the livers of PD-challenged mice (Fig. 2A). Such areas were neither detected in the control livers (Fig. 2A) nor in the LPS-challenged animals (not shown). Color-coded mean velocity maps showed a decrease in the number of blood vessels in PD-challenged livers (Fig. 2A; white triangles). Yellow was used to indicate the highest, and red - the lowest speed of blood flow. Also, the impaired hepatic blood flow, reflected in a low content of yellow/orange, was observed in the PD-challenged mice (Fig. 2A). In contrast, no significant velocity changes (such as decreased speed of blood flow and resulting perfusion abnormalities) were revealed in the livers of the LPS-challenged animals. The 3D computational models of the liver vasculature in the control, LPS- or PD-challenged mice (Fig. 2B; upper, middle and bottom models, respectively) showed differences in the hepatic vasculature even more clearly, which was supported by a further analysis of the vascular volume (Fig. 2C). 3D modelling analysis confirmed a ~75% reduction in the liver vascular volume in PD-challenged animals (cream bar), compared to the control (orange bar).

### 4.2 Photodynamic challenge in the liver leads to necrotic tissue damage

Then we characterized liver tissue by (immuno)histological analysis. Paraffin-embedded tissue samples of all experimental animals and frozen liver tissue samples of PD-challenged mice, were examined in detail (Fig. 3). Histological staining methods, i.e. hematoxylin & eosin (HE; Fig. 3A, C, E, G) and May-Grünwald-Giemsa (MGG; Fig. 3B, D, F, H), demonstrated the presence of necrotic areas of the liver parenchyma solely in PD-challenged mice (Fig. 3E–H; see also macrophotographs Fig. 3I–J). These necrotic areas were distinguished from normal parts by their distinctive discoloration (pale yellow color instead of brown native to the liver) and were later identified as being of a purely ischemic nature. Ischemic tissue contained predominantly dead hepatocytes of “translucent” appearance and they stained uniformly with eosin, owing to the dissolution of nuclei and loss of intercellular borders (Fig. 3E). The necrotic zones, as seen in the staining (Fig. 3G–H; red arrows), were infiltrated by mononuclear leukocytes of various types; the presence of extravasated blood was also detected in the necrotic liver parenchyma (Fig. 3E–H; blue arrows). Further immunohistochemical staining for the endothelial marker CD31 was carried out to compare the lumen patterns (vascular, not ductal) in the normal versus necrotic tissue parts of the PD-challenged livers (Fig. 3K-L; yellow arrows). While the normal hepatic parenchyma contained fully functional blood vessels (Fig. 3K), collapsed vessels in the ischemic parts (Fig. 3L) were surrounded by a prominent tissue scar, and were unable to transport blood effectively (see also Fig. 2B–C). The boundaries between the normal and ischemic tissues were infiltrated by the CD107b-expressing mononuclear phagocytotic cells (Fig. 3M; red arrows), likely the macrophages.

### 4.3 Photodynamically challenged liver contains areas of intensive reactive and regenerative processes

In order to provide further insights into cellular components of the PD-challenged livers, we used confocal microscopy and fluorochrome-conjugated antibodies (Fig. 4). The cells present in the scar tissue between the normal and ischemic zones were probed simultaneously for the presence of the pan-leukocyte marker CD45 (Fig. 4A, C, E-F) and the myofibroblast-like cell marker α-smooth muscle actin (α-SMA) (Fig. 4A, D, F-G). The nuclei were counterstained with DAPI (Fig. 4A–B, E, G). The CD45-expressing cells infiltrated mainly the areas adjacent to the blood vessels, but were also scattered in the scar tissue, including its edges (Fig. 4A, C, E-F). The α-SMA-expressing cells, likely hepatic stellate cells [39], could only be found in the areas of insult, in which they formed a discrete network of elongated cells spreading toward the ischemic tissue (Fig. 4A, D, F-G). The presence of CD45- or α-SMA-expressing cells, respectively, indicated the dynamic nature of the reactive (here: inflammatory) or regenerative processes (fibrosis development) in the boundaries between the necrotic and live areas of the tissue in the close vicinity of blood vessels.

### 4.4 Stress affects the EPR spectra of transition metal complexes *in situ*

We used EPR spectroscopy to analyse the snap-frozen liver or blood samples at 77 K, which detects paramagnetic species, including metal complexes in biological samples. This analysis revealed differences in the *in situ* signals recorded in liver and whole blood samples (Fig. 5; see also Table 2). The EPR spectra of the intact (the “No spin trap” group of mice that is free from any exogenous spin trap) control samples of both liver and blood (Fig. 5A) showed paramagnetic components like those of the semiquinone, molybdenum(V) or reduced iron-sulfur clusters, whereas the blood samples also showed the presence of NO-hemoglobin adduct (nitrosylhemoglobin, HbNO). The HbNO signal contained a component of a narrow triplet hyperfine structure (HFS; blue marker). To detect free radicals in other liver and blood samples, the Fe^II^-DETC complexes were used as exogenous spin traps (Fig. 5B–F). Compared to the intact control (Fig. 5A, left), the Fe^II^-DETC complexes did not re-shape the spectra recorded in the vehicle (Fig. 5B, left), vehicle and light (Fig. 5C, left) and Zn-Pheide and no-light (Fig. 5D, left) control liver groups. Interestingly, in the blood samples collected from these groups of animals, a background signal of Cu^II^(DETC)_2_ complexes that contained a component of a quarter HFS was found (Fig. 5B–D, right; red marker). In the blood samples, the Cu^II^(DETC)_2_ HFS (red pointers, line III) partially overlapped with the HbNO HFS (blue pointers, line II). The normal part of the PD-challenged livers (Fig. 5E, left; black solid line) revealed spectral characteristics similar to those of the controls (Fig. 5B–D). This was in sharp contrast to the ischemic part of the PD-challenged livers (Fig. 5E, left; grey solid line) in which all typical paramagnetic components of the liver spectrum (the semiquinone free radical, Mo(V) or reduced iron-sulfur clusters), but a marked Cu^II^(DETC)_2_ signal, were absent. Since Cu^II^(DETC)_2_ was the only EPR-detectable species, this indicates a dramatic change in the paramagnetic centers of the liver tissue that become EPR-silent (quenched). In general, the Cu^II^(DETC)_2_ signals in the blood were of a much lower amplitude, and the EPR spectrum did not change upon PD-challenge (Fig. 5E, right), whereas endotoxemic stress changed both the liver and blood EPR patterns (Fig. 5F). Their spectra contained typical EPR signals of paramagnetic NO–Fe^II^(DETC)_2_ complexes of a narrow triplet HFS (green marker), which confirms NO production. Therefore, in our experimental settings, LPS-challenge also provides a positive control for photodynamically stressed livers.

### 4.5 Stress affects NO level in the liver and blood

We investigated the presence of NO in the liver and blood samples by EPR spin trapping in the form of stable paramagnetic spin NO adducts (Fig. 6). Fe^II^-DETC complexes and hemoglobin served as exogenous and endogenous NO spin traps, respectively. A significant increase in the NO levels (p = 0.008), as compared to the control, was observed in the livers after LPS challenge (see variations at the amplitude of NO–Fe^II^(DETC)_2_ signals; line III, Figs. 5F and 6A). In contrast, the PD-challenge dramatically decreased (p = 0.003) the NO levels in the ischemic part of the liver (filled circles), but it did not affect the normal part (empty circles) of this organ (Fig. 6A). Amplitudes of the NO–Fe^II^(DETC)_2_ signals were also assessed in the blood samples (Fig. 6B), where a significant increase in NO levels (p = 0.012) was found solely in LPS-challenged animals. Analysis of hemoglobin-trapped (HbNO), and thus “bio-available” NO, confirmed an elevation in its level in the blood (line I, empty squares; and line III, filled squares in Fig. 6C; see also Fig. 5). Again, LPS-challenged animals had higher blood NO levels than mice in other experimental groups.

### 4.6 Paramagnetic Cu content soars in the liver upon photodynamic challenge

Analyzing paramagnetic Cu complexes in the liver and blood shows that only the ischemic livers were the source of the Cu(II) spin adduct to DETC, Cu^II^(DETC)_2_ (Fig. 7A; see also Fig. 5, left). Unlike any other analyzed liver tissue (empty circles, Fig. 7A), an increase in the Cu(II) signal (amplitude of line III; filled circles, Fig. 7A) in the ischemic PD-challenged liver was significant (p = 0.002). In the blood samples, the Cu^II^(DETC)_2_ signals had similar levels in all experimental groups (amplitudes of line III, filled squares; and line IV, empty squares Fig. 7B).

### 4.7 Stress induces elevations in liver and blood paramagnetic Fe species

We used EPR spectroscopy to detect the paramagnetic Fe complexes in the liver and blood samples. Reduced Riske cluster (Fe–S) EPR signals were present in all but the ischemic parts of the PD-challenged livers (filled circles, Fig. 8A). The drop in the Fe–S signal was significant (p = 0.002) as compared to the normal part of the PD-challenged livers or controls (empty circles, Fig. 8A). Similarly to the Fe–S signals, the EPR spectroscopic signals of Mo(V) were absent in the ischemic parts of the PD-challenged livers (not shown). Other paramagnetic Fe species, such as Fe(III) in methemoglobin (MetHb), or transferin-bound Fe (TF), were also seen in the blood (Fig. 8B–C). Elevated MetHb levels were detected in the blood samples collected from animals that had undergone PD/LPS challenge, but not in control animals (Fig. 8B). However, in blood samples from the control and PD-challenged mice the Fe^III^-TF levels were similar and slightly decreased in LPS-challenged animals (Fig. 8C).

## 5 Discussion and conclusions

Although micronutrients such as Cu, Fe and others have long been acknowledged to be redox cofactors in fundamental physiological reactions, biologists tend to perceive them merely as elements of inorganic chemistry. Nevertheless, recent extensive studies on these metals in cells and tissues provide new insights into the roles of redox-active trace elements in physiological and pathological processes. However, understanding of the actual involvement of transition metal ions accumulated in the body (and hence potentially harmful) in the mechanisms of pathology is still very limited. Since Fe ions have recently been identified as the regulators of ferroptosis (a unique non-apoptotic cell death) [12] it may be anticipated that further studies will also reveal the roles of other transition metals in a variety of biological processes [6]. For example, Cu may modulate the function of synaptic receptors and channels, being also implicated in severe neurodegenerative disorders [6,40]. Significantly, disruption in tissue copper homeostasis, which has been studied in mice with Cu transporter cardiac-specific knockout, may be communicated across organ systems [41,42]. In these knockout animals a “low Cu” signal carried from the heart via blood serum mobilized Cu from its hepatic and intestinal supply. Also, other studies have shown that both Cu and Fe levels increase in the case of post-ischemic heart injury [43]. In the light of these findings, the subject of this study - determining whether under pathological conditions redox-active transition metals are recruited from the liver (a key organ for storing Cu and Fe) to the blood - is of particular importance. However, photodynamic challenge, but not LPS administration, affect hepatic vasculature, leading to ischemic necrosis in the liver parenchyma, as evidenced by ultrasound examination, 3D computational modelling (Fig. 2), and histological staining (Fig. 3), respectively. This has prompted us to extend our study and investigate the putative involvement of nutritional transition elements in the processes that drive liver necrotization under a limited supply of O_2_ (and thus a lower probability of electron exchange with O_2_). The development of necrotic damage in this organ, as a result of local photosensitization (treatment with a light-activated pro-drug in the presence of light), has been previously reported by us [24] and others [44,45]. These studies, however, have not provided spectroscopic or (immuno)histological insights into the nature of hepatic necrosis, which is of potential clinical relevance, e.g. in liver transplantation. The present study demonstrates not only that PD-challenge reduces blood supply to the tissue (Fig. 2; see also Fig. 3H, K-L), but also delivers a detailed description of the cellular components of the necrotic liver (Figs. 3 and 4). It is noteworthy that histological analysis of the septic liver, achieved by a non-lethal dose of LPS administered 24 h before the sample collection, did not reveal any differences from the control tissues (Fig. 3A–D), whereas PD-challenge resulted in the development of the foci of hypoperfused tissue (Fig. 3E–J). Further analyses of the necrotic tissue confirmed the presence of live cell populations close to the hollow cavities (lumens of blood vessels or ducts). Via the expression of CD31 (Fig. 3L) in the endothelium, these have been identified as blood vessels (ductal epithelium lacks CD31 expression) [46,47]. This resembles a typical liver regeneration pattern outlined by intensive processes of tissue restoration, as performed by the cells that fuel a desmoplastic reaction and reactive processes, as driven by immune cells (Fig. 3G–H, M and Fig. 4), both processes being confined to vessels/ducts [48]. CD107b-positive mononuclear phagocytes, likely macrophages, were found in necrotic areas in the vicinity of blood vessels (Fig. 3M). Importantly, macrophages have commonly been acknowledged for their regulatory role in systemic Fe homeostasis [49]. Therefore, the abundance of them in the PD-challenged tissue may be attracted by Fe-rich dead cells, such as erythrocytes from the extravasated blood, or hepatocytes (Fig. 3G–H). By removing cell debris, macrophages prevent Fe depletion and maintain its homeostasis. The presence of other immune cells, the CD45-positive leukocytes, in close proximity to the α-SMA-expressing cells, is probably caused by a desmoplastic reaction that secures tissue integrity in the injured organ (Fig. 4). The α-SMA-positive cells, likely hepatic stellate cells (HSCs) [39], produce extracellular fibers to restore tissue mechanostasis and thus provide advantageous conditions for liver regeneration [50]. Notably, the processes of HSC activation and production of the fibrous matrix may become accelerated by Fe overload-induced oxidant stress [14]. Furthermore, complex tissue-healing processes (especially in their early phases) may be facilitated via the production of NO by infiltrating immune cells (e.g. macrophages) and stellate cells [33,51,52]. We therefore attempted to determine the plausible involvement of NO in the processes ongoing in the PD-challenged livers, and compare these processes to those observed in the LPS-challenged organ [27,53]. In our experimental strategy of NO-spin trapping we have implemented recommendations of Vanin and Kleschyov [35], who gave the DETC-injected animals exogenous Fe. Noteworthy, the same authors reported that even the physiological amount of Fe(II) in mouse body fluids is sufficient to form Fe-DETC complexes fully capable of NO trapping [35]. This also means that an experimental group receiving DETC only (no exogenous Fe(II) supplementation; not examined in our study) would still be able to generate an active form of the spin trap, although to a lesser extent, using the endogenous Fe(II) pool [34,35]. In a form of such complexes, NO has been detected by EPR spectroscopy at liquid nitrogen temperatures. One of the main advantages of this method is its excellent specificity in detecting the paramagnetic species, including certain transition metal ions present in the biological samples [54]. Indeed, the liver and blood EPR spectra contain components originating from transition metal ions/complexes. These included copper(II) diethyldithiocarbamate complexes, Cu^II^(DETC)_2_; molybdenum(V), and the reduced Riske cluster, Fe–S, in the liver along with methemoglobin, MetHb; transferrin-bound iron(III), Fe^III^-TF; copper(II) diethyldithiocarbamate complexes, Cu^II^(DETC)_2_, and nitrosylhemoglobin, HbNO, in the blood (Fig. 5 and Table 2). The EPR spectra of all the control livers and necrosis-free parts of the PD-challenged organs are similar (Fig. 5A–E) while those of the necrotic livers (Fig. 5E) contain a marked component of the Cu^II^(DETC)_2_ signal, lacking virtually all other EPR signals that are normally seen in this organ. Notably, these dramatic differences occur within the same organ between sections harvested from its live and dead zones (Fig. 5E; see also Figs. 7A and 8A). Importantly, diethyldithiocarbamate used here as a spin trap has a potent copper-chelating capacity [55], yielding Cu(II)-DETC complexes that are more stable than other DETC complexes with divalent metal ions, for example Fe(II) [56]. This strong affinity of DETC for Cu opens up a potential therapeutic avenue for Menkes syndrome (neurodegenerative Cu deficiency). Patients suffering from this disorder could be given Cu^II^(DETC)_2_ complexes orally in order to increase their Cu levels towards more physiological values [55]. Other EPR signals of paramagnetic DETC complexes, NO–Fe^II^(DETC)_2_ (Fig. 5F), characterized by a narrow triplet hyperfine structure, were detected clearly in LPS-challenged liver (p = 0.008 vs control) and blood (p = 0.012 vs control) samples, but not in other groups (Fig. 6A–B). Similarly, the signal from another paramagnetic NO adduct, HbNO, was elevated solely in the blood collected from endotoxemic animals (Fig. 6C). According to the historic dogma, HbNO had been a hallmark of necrosis: NO released by inflammatory cells infiltrating the necrotic tissue is trapped by hemoglobin form extravasated blood, yielding HbNO [57,58]. The absence of HbNO signal in the EPR spectra of necrotic liver (Fig. 5E) might be explained by the ischemic (shortage of blood) and not hemorrhagic (massive blood extravasation) character of necrosis. Further, in terms of NO content, the ischemic liver contained much less NO–Fe^II^(DETC)_2_ than the non-necrotic liver zones (p = 0.003; Fig. 6A), despite the ongoing reactive/regenerative processes (Figs. 3 and 4). This may be explained by the rather modest live/necrotic cell ratio in this tissue. Finally, the reduced Riske cluster signals in necrotic tissue were almost undetectable (p = 0.002 vs control/normal part of a PD-challenged liver, Fig. 8A). Taken together, our results indicate that at least some of the physiologically paramagnetic species (detectable by EPR spectroscopy) undergo redox reactions during ischemia development, yielding diamagnetic (EPR-silent) species, or a mix of those. Another possible reason is the lack of endogenous reductants in the ischemic tissue which could convert the diamagnetic NO–Fe^III^-(DETC)_2_ adducts into paramagnetic NO–Fe^II^-(DETC)_2_ ones [59]. This, however, does not explain the presence of strong Cu^II^(DETC)_2_ signals (p = 0.002 vs other samples, regardless of the treatment) in the ischemic zones (Figs. 5E and 7A). A strong Cu(II) signal in the EPR spectra of ischemic livers may originate from a photodynamic Cu oxidation *in situ* in metal-storing liver cells (hepatocytes and others) as opposed to ischemia-elicited interconversion of intrinsic hepatic Cu(I), bound to copper chaperones, to Cu(II). Under physiological conditions, cooper ions predominantly exist as diamagnetic (EPR-silent) Cu(I) [3,54,60], and in pathophysiological conditions may be further oxidized to Cu(II). Copper(II) is then complexed with a spin trap, yielding a paramagnetic species Cu^II^(DETC)_2_ of a very high stability, much higher than this of Fe^II^-(DETC)_2_ [56]. Those Cu complexes are easily detectable by EPR spectroscopy, especially in a hydrophobic membrane environment [61]. Importantly, due to the catalytic activity of Cu(II) harmful lipid-based radicals may be produced in the body [11], and indeed Cu(II)-loading has been reported to worsen post-ischemic injury of the heart [62]. As such, in order to prevent exacerbation of Cu-mediated radical reactions, Cu(II), upon being delivered to the target tissue, needs to be either reduced back to Cu(I) and transported to target proteins [3], or removed from the body. Since Cu is transported within the body as Cu(II), the presence of Cu^II^(DETC)_2_ may enhance copper excretion with bile, which is a typical means of copper clearance [[3], [4], [5]]. Additionally, only a modest increase in Cu(II) levels in the blood (Figs. 5 and 6B) indicates a lack of Cu recruitment across the body from its hepatic store, pointing towards its reduction back to Cu(I) and/or excretion with bile. However, analytical measurements of biliary Cu (as well as other transition elements) are out of the scope of this study. It needs to be acknowledged, however, that the use of a spin trap DETC makes it impossible to detect a serum Cu-transporting protein ceruloplasmin (CP) using EPR. By contrast, in DETC-free samples, CP can be easily detected by EPR owing to its broad singlet signal at g = 2.05. Importantly, CP level soars in severe inflammatory processes that underlie the septic shock or advanced stages of cancer, and EPR is an appropriate method for studying these rises in CP. Nevertheless, our earlier studies on blood samples (DETC-free) collected from the mice that received Zn-Pheide and light therapy to the lung xenograft tumors or PD-stress to the liver, revealed no changes in CP levels upon the photodynamic treatment [24]. Therefore, in this study, we did not focus on studying CP in the blood.

Nevertheless, apart from the elevated NO levels (Fig. 6B–C), at least a few spectral “fingerprints” of stress have been assessed in blood samples. First, increases in methemoglobin content, pointing toward the oxidation of heme iron into Fe(III), have been detected in blood samples collected from the PD- or LPS-challenged animals (Fig. 8B). A similar elevation in MetHb upon stress, also assessed using EPR spectroscopy, has been reported in human subjects, who having worked in 1986 as liquidators of the Chernobyl disaster, became exposed to low-intensity radiation [63]. Another stress-related change in the paramagnetic “landscape” of the blood spectra analyzed is a decrease in transferrin-bound iron(III) in endotoxemic animals (Figs. 5 and 8C). This notion is in line with the findings of so called “anemia of inflammation” - which is essentially limited Fe availability upon infection that acts to decrease microbial pathogenicity [64]. Anemia of inflammation also occurs in neoplastic diseases, and this may explain decreases in Fe^III^-TF in the blood of the liquidators mentioned above who likely developed neoplasia as a result of exposure to radiation [63].

Taken together, here we establish a new model of hepatic ischemia. The model of ischemic induction proposed by us is relatively easy to achieve - it neither requires specialist training, nor complicated procedures or advanced equipment. Even more importantly, the model involves only minimal animal distress as compared to the surgical models. In our model, we show that on the cellular and sub-cellular levels ischemic liver tissue differs dramatically from normal hepatic parenchyma, as confirmed by microscopic and spectroscopic techniques. On the basis of EPR measurements, we also hypothesize about the role that redox-active transition metals play in liver health and pathophysiology. These results are important for an understanding of the processes that drive the development of liver necrosis, which is very relevant to organ transplantation and injury.

## Declaration of competing interest

None.
